# Clinical evaluation of the bed cycling test

**DOI:** 10.1002/brb3.445

**Published:** 2016-04-08

**Authors:** Katharina Feil, Nicolina Boettcher, Franziska Lezius, Maximilian Habs, Tobias Hoegen, Katrin Huettemann, Carolin Muth, Ozan Eren, Florian Schoeberl, Andreas Zwergal, Otmar Bayer, Michael Strupp

**Affiliations:** ^1^ Department of NeurologyUniversity HospitalMunichGermany; ^2^ German Center for Vertigo and Balance DisordersUniversity HospitalMunichGermany; ^3^ Department of Anesthesiology and Operative Intensive Care MedicineCharité UniversityBerlinGermany

**Keywords:** Cerebrovascular disease/Stroke, clinical neurology, clinical neurology examination

## Abstract

**Objective:**

Additionally to the forearm rolling test to detect mild unilateral upper limb dysfunction, the bed cycling test (BCT) for detection of mild to moderate lower limb dysfunction was developed, evaluated and compared to the leg holding test.

**Methods:**

In a prospective observer‐blinded study, 60 patients with MRI/CT‐proven focal cerebral hemisphere lesions and a mild to moderate unilateral paresis of the lower limb (graduated MRC 3–4/5), and 60 control persons with normal imaging were examined and filmed. Nine observers blinded to the diagnosis evaluated these videos. The sensitivity, specificity and the positive and negative predictive values of the clinical tests were analyzed.

**Results:**

The observers gave a correct evaluation of BCT in 35.5% of all patients with focal cerebral lesions compared to 26.0% for the leg holding test. On the other hand, observers had false negative results in 29.1% of cases with BCT and 44.7% with leg holding test. In 36.7% of patients, only BCT was pathological while leg holding test was unremarkable. The sensitivity of the combination of both tests was 0.68 (95% CI 0.61–0.75). The BCT is more sensitive (64.3%) than leg holding test (46.2%) while the specificity of leg holding test (85.6%) is higher than of BCT (70.1%) to detect a cerebral lesion affecting the lower limb. The inter‐rater variability is high with no differences comparing different types of clinical experience.

**Conclusions:**

The BCT is a useful additional clinical bedside test to detect subtle unilateral cerebral lesions. The BCT is easy to perform and can be added to the routine neurological examination.

## Introduction

There is a variety of clinical tests that diagnose a cerebral lesion affecting the upper and lower limb by identifying limb paresis as a sign of unilateral focal cerebral lesion (Anderson et al. 2005). Pronator drift of the upper limb and the holding test of the upper (Barré) and lower limb (Mingazzini maneuver) are the most common tests (Koehler 2000). In recent years, the sensitivity and specificity of different clinical tests were systematically investigated, focusing on clinical tests of the upper limb by several different studies (Teitelbaum et al. 2002; Amer et al. 2012).

In previous studies, the implementation of two simple bedside tests, namely, the forearm rolling test and the finger rolling test in addition to known clinical tests (e.g., arm holding experiment, wrist extension, tendon reflexes, finger tapping) increased the sensitivity and specificity of the identification of focal cerebral lesions (Sawyer et al. 1993; Ko and Verhagen 1994; Yamamoto 1995; Teitelbaum et al. 2002; Amer et al. 2012). For instance, a three test combination consisting of pronator drift, finger tap and deep tendon reflexes led to a sensitivity of 97.8% and a negative predictive value of 95.5% for a central motor lesion. Two variants of the forearm rolling test are the index finger rolling test (Yamamoto 1995) and the thumb rolling test (Nowak 2011). All these additional tests assess rapid alternating movements of more proximal muscles of the arm and shoulder. Based on the distal to proximal gradient of the cortico‐spinal system, a test assessing more distal muscles of the hand is assumed to be more sensitive to detect subtle impairments of the cerebral cortico‐spinal tract when other clinical tests are normal.

Comparably to those validated tests of upper limb function we now developed and evaluated a new clinical bedside test to detect unilateral lower limb dysfunction: the “bed cycling test” (BCT). In an observer‐blinded prospective study using videos, we analyzed the test's sensitivity and specificity and compared it to the leg holding test (LHT) in the supine position.

## Methods

### Study protocol

All patients and control subjects underwent a detailed standardized clinical examination by experienced neurologists. The following five tests were performed and documented using videos: (1) BCT forwards; (2) BCT backwards; (3) leg holding test; (4) Babinski's sign; and (5) 5‐m walking test.

**Figure 1:**
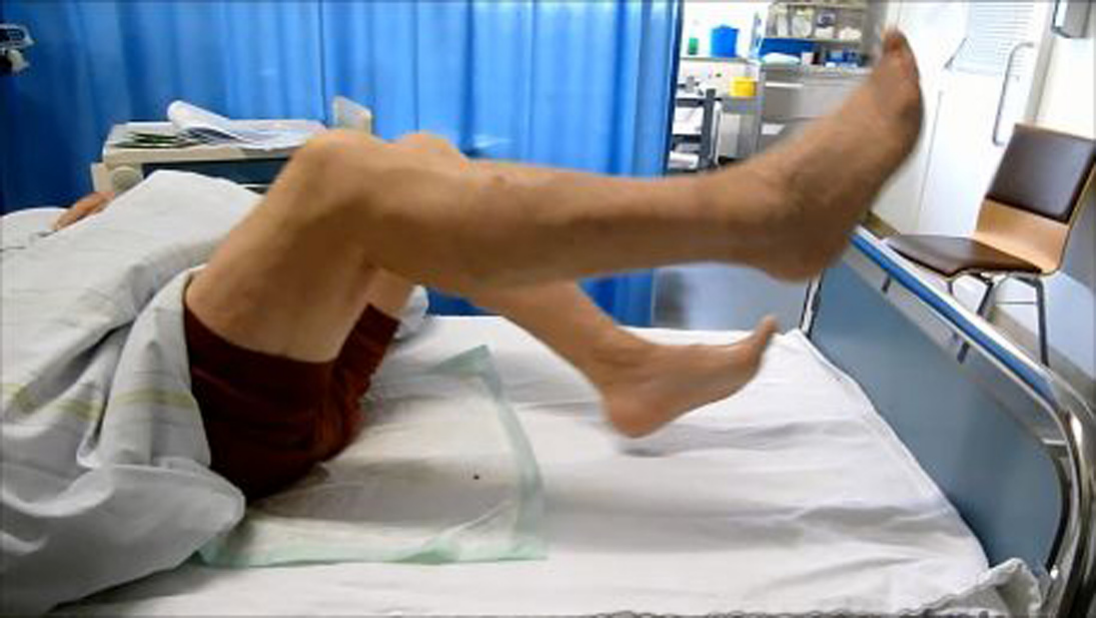
Example of a patient performing the bed cycling test (BCT) (patient no. 5). Patient is lying on his back. Both legs are flexed in the hip. The patient is asked to air cycle rapidly with eyes closed forwards for 10 sec and backwards for 10 sec (so called “bed cycling”).

The BCT was performed by asking the patients or healthy control persons to close their eyes and pedal with their legs in the air backwards for 10 sec and forwards for 10 sec (see Fig. 1). The BCT was documented as pathological when the leg of the affected lower limb rotated significantly slower and was less coordinated or even remained stationary while the unaffected leg moved normally. This suggested a unilateral central lesion. The bed cycling test – forwards and backwards – was rated together as one result (pathological or non‐pathological).

The LHT in the supine position was tested by asking the patients and healthy control subjects to hold both legs at an angle of 90° flexed at the hip and knees for 10 sec with eyes closed. Muscle strength was graded out of five using the Medical Research Council (MRC) scale (Palo Alto 1978; Paternostro‐Sluga et al. 2008).

### Randomization and allocation to groups and rating videos

There were three videos of each study patient: one video showing only the bed cycling test (BCT), one video showing only the LHT and one video containing both clinical tests (the BCT and the LHT) resulting in 360 different videos. The 120 study participants were randomly assigned to three groups of 40 patients each. From this material we compiled the three rating videos, each consisting of BCT videos from one, LHT videos from another, and BCT + LHT videos from the remaining randomized group, as shown in Table 1. Each rating video was assessed by three neurologists with different grades of experience ranging from 0.5 to 20 years. Therefore, there were nine raters judging the videos. All of the raters were naive regarding medical history and clinical, laboratory and imaging data of each patient or control. Based on the videos the raters had to decide, if there was a paresis, on which side and of which grade (5, 4+, 4, 3). The videos were shown to each physician independently and in isolation.

**Table 1:** Compilation of rating videos for analysis

	Rating video no. 1	Rating video no. 2	Rating video no. 3
BCT	Group 1	Group 2	Group 3
LHT	Group 2	Group 3	Group 1
BCT + LHT	Group 3	Group 1	Group 2

BCT, bed cycling test; LHT, leg holding test in supine position.

Each participant was assigned to the corresponding group by screening for study, according to the inclusion and exclusion criteria. Furthermore, the patients were examined and the grade of paresis was specified according to clinical criteria. The study was conducted prospectively with a recruitment period of 30 months (2011–2014).

### Patient group

Subjects met the following inclusion criteria: (1) age ≥ 18 years; (2) evidence of an unilateral focal lesion in the computed tomography (CT) or magnetic resonance imaging (MRI); (3) paresis of the lower extremity with a muscle strength grade ≥ 3/5. Patients with the following criteria were excluded: (1) age < 18 years; (2) imaging showed lesions in the cerebellum or diffuse cerebral lesions; (3) patients with preexisting gait disturbance; (4) patients with disturbance of mobility/strength of the limb due to other and/or preexisting causes; (5) patients showing paresis of the lower extremity with a muscle strength grade (MRC grade) < 3/5; and (6) patients who were not able to give their consent in the study design.

### Control group

Healthy control persons were recruited in the German Center for Vertigo and Balance disorders and in the neurological clinic. Clinical routine imaging had to exclude a focal cerebral lesion (by CT or MRI). Exclusion criteria for the control group were the following: (1) age < 18 years old; (2) Patients who were not able to give their consent in the study design; (3) preexisting gait disturbance; and (4) preexisting disturbance of mobility/strength of the extremities.

### Ethic standard protocol approvals, registration and patient consent

Approval from the ethics committee board of the University of Munich was obtained for the study. Written informed consent was obtained from all participants (controls and patients). All clinical investigations were conducted according to the principles of the Declaration of Helsinki. Every patient and control person confirmed the use of the anonymized videos for analysis and publication.

### Statistical analysis

Data were collected using Excel (Microsoft, Redmond, WA) spreadsheet software. Statistical analysis was performed using R version 3.1 (www.r-project.org) with StatWeave (homepage.stat.uiowa.edu/~rlenth/StatWeave/). The sensitivity, specificity, positive and negative predictive values (with exact confidence intervals) and likelihood ratios were calculated for each test. Differences were considered significant if *P* < 0.05.

To assess inter‐rater reliability, Poisson regression was used to model the contingency tables between the three raters of each group with no paresis, left paresis, or right paresis as possible rating options. One additional parameter for the diagonal (representing agreement) was added, as a result of the model selection procedure using likelihood ratio tests. Since the three rater groups viewed different videos, one model was used for each rater group.

To assess the effect of rater experience, MRC grade, and test we modeled the odds to obtain a correct result (i.e., true positive or true negative) using a random effects logistic regression model (Bates D, Maechler M, Bolker B and Walker S [2014]. lme4: Linear mixed‐effects models using Eigen and S4_. R package version 1.1‐7, http://CRAN.R-project.org/package=lme4) with a random intercept per patient, the confidence intervals were estimated using a parametric bootstrap.

## Results

### Patient group

Sixty‐five patients were included, five had to be excluded for technical reasons. Sixty consecutive patients (26 females, mean age 68.4 ± 14.4 years) were examined. Clinical characteristics of the patients, including the details of the neurological examination are summarized in Tables 2 and 3. Most of the patients (98.3%) had symptoms due to an acute event (ischemia or hemorrhage), only one patient was suffering from a tumor. Eighty percent of lesions were located supratentorially, mostly represented by the mid cerebral artery territory. Supplemental material online only shows patient no. 5 with a paresis on the right site (Video S2) and patient no. 40 with a paresis on the left site (Video S3). For more detailed information about the anatomical localization of the lesion, see Table 4.

**Table 2:** Summary of clinical data of patients

Clinical data of patients	Total number of patients
Etiology	
Ischemia	56
Hemorrhage	3
Tumor	1
Lesion location	
MCA	43
ACA	3
PCA	2
Mesencephalon	5
Pons	5
Medulla oblongata	1
Side of lesion	
Left	24
Right	36
Onset of symptoms	
Up to 72 h	29
72 h up to 3 months	24
More than 3 months	7
Graduation of paresis (muscle strength grade)	
3/5	10
4/5	50
Leg holding test in supine position	
Pathological	38 (17 left/21 right)
Not pathological	20
Both sides/not evaluable	0
Bed cycling test forwards	
Pathological	57 (31 left/26 right)
Not pathological	3
Both sides/not evaluable	0
Bed cycling test backwards	
Pathological	56 (32 left/24 right)
Not pathological	2
Both sides/not evaluable	0
Heel‐knee test	
Pathological	39 (19 left/20 right)
Not pathological	15
Both sides/not evaluable	0
5‐m walk	
Pathological	36
Not pathological	9
Both sides/not evaluable	15 (not feasible/patient not able to walk)
Muscle tone lower limb	
Pathological	5 (1 left/4 right)
Not pathological	54
Both sides/not evaluable	0
Reflexes	
Pathological	6
Not pathological	54
Both sides/not evaluable	0
Babinksi's sign	
Pathological	16 (8 left/8 right)
Not pathological	44
Both sides/not evaluable	0
Sensibility	
Pathological	14
Not pathological	46
Both sides/not evaluable	0

MCA, mid cerebral artery; ACA, anterior cerebral artery; PCA, posterior cerebral artery.

**Table 3:** Clinical data of patients

Patient no.	Age	Sex	Onset of symptoms	Etiology	Lesion location	Side of lesion	Graduation of paresis	LHT	BCT forwards	BCT backwards	Babinski sign	5‐m walk	Sensibility	Reflexes	Heel‐knee test
1	81	Male	72 ho up to 3 months	1	1	Right	4	+ left	+ left	+ left	−	+	−	−	+ left
2	55	Male	Up to 72 h	1	1	Right	3	+ left	+ left	+ left	+ left	Not feasible	+	+	+ left
3	73	Female	Up to 72 h	1	1	Left	3	+ right	+ right	+ right	+ right	+	−	−	+ right
4	82	Female	Up to 72 h	1	1	Right	4	+ left	+ left	+ left	−	+	−	−	+ left
5	82	Male	Up to 72 h	1	1	Left	4	−	−	+ right	−	−	−	−	+ right
6	92	Female	72 h up to 3 months	1	1	Right	4	+ left	+ left	+ left	+ left	+	−	−	+ left
7	73	Female	72 h up to 3 months	1	1	Right	3	−/+ both	+ left	+ left	+ left	Not feasible	+	+	Not feasible
8	66	Female	72 h up to 3 months	1	1	Right	4	+ left	+ left	+ left	−	+	+	−	+ left
9	54	Female	72 h up to 3 months	1	1	Left	4	+ right	+ right	+ right	−	+	−	−	−
10	78	Female	72 h up to 3 months	2	4	Right	3	+ left	+ left	+ left	−	Not feasible	−	−	Not feasible
11	62	Male	72 h up to 3 months	1	1	Right	4	+ left	+ left	+ left	−	+	−	−	+ left
12	79	Female	72 h up to 3 months	1	5	Right	4	+ right	+ right	+ right	−	+	+	−	+ right
13	71	Male	72 h up to 3 months	1	1	Left	4	−	+ right	+ right	+ right	+	−	−	+ right
14	47	Male	Up to 72 h	1	1	Left	4	−	+ right	+ right	+ right	−	−	−	+ right
15	55	Male	72 h up to 3 months	1	5	Left	4	+ right	+ right	+ right	+ right	+	+	−	+ right
16	86	Female	Up to 72 h	1	1	Left	4	+ right	+ right	+ right	−	+	−	−	+ right
17	78	Female	72 h up to 3 months	1	1	Right	4	+ left	+ left	+ left	−	+	−	−	−
18	62	Male	72 h up to 3 months	2	1	Left	3	+ right	+ right	+ right	−	Not feasible	−	−	+ right
19	72	Female	72 h up to 3 months	1	1	Left	4	+ right	+ right	+ right	+ right	+	−	−	−
20	87	Female	72 h up to 3 months	1	1	Right	4	+ left	+ left	+ left	−−	+	−	−	−
21	52	Male	Up to 72 h	2	4	Right	4	+ left	+ left	+ left	−	Not feasible	+	+	+ left
22	38	Male	72 h up to 3 months	1	1	Right	4	−	+ left	+ left	+ left	−	−	−	+ left
23	69	Male	72 h up to 3 months	1	3	Right	4	−	−	+ left	−	−	−	−	−
24	54	Male	72 h up to 3 months	1	5	Left	4	+ right	+ right	+ right	+ right	+	−	−	+ right
25	68	Male	Up to 72 h	1	1	Left	4	+ right	+ right	+ right	+ right	+	−	−	+ right
26	78	Female	Up to 72 h	1	1	Left	4	+ right	+ right	+ right	−	+	−	−	+ right
27	73	Female	72 h up to 3 months	1	1	Right	3	+ left	+ left	+ left	−	+	−	−	+ left
28	80	Female	72 h up to 3 months	1	1	Left	4	+ right	+ right	+ right	−	+	−	−	+ right
29	92	Male	Up to 72 h	1	1	Right	3	−	+ left	+ left	−	+	+	−	+ left
30	81	Male	72 h up to 3 months	1	4	Left	4	+ right	+ right	+ right	−	Not feasible	−	−	+ right
31	34	Female	More than 3 months	1	6	Right	4	+ right	+ right	−	−	−	+	−	−
32	68	Female	Up to 72 h	1	1	Left	4	+ right	+ right	−	−	Not feasible	−	−	Not feasible
33	68	Male	72 h up to 3 months	1	2	Left	4	+ right	+ right	+ right	−	+	−	−	+ right
34	74	Male	More than 3 months	1	1	Left	4	+ right	+ right	+ right	−	+	−	+	+ right
35	80	Female	72 h up to 3 months	1	1	Right	4	−	+ left	+ left	−	+	−	−	+ left
36	93	Male	Up to 72 h	1	1	Right	4	+ left	+ left	+ left	+ left	+	−	−	+ left
37	68	Female	Up to 72 h	1	2	Left	4	+ right	+ right	−/+ both	−	Not feasible	−	−	−
38	75	Male	Up to 72 h	1	1	Right	4	−	+ left	+ left	−	−	−	−	−
39	81	Male	Up to 72 h	1	1	Right	3	−	+ left	+ left	+ left	+	−	−	+ left
40	21	Male	More than 3 months	3	1	Right	4	−	+ left	+ left	−	+	−	−	+ left
41	72	Male	More than 3 months	1	1	Left	4	+ right	+ right	+ right	−	+	+	+	+ right
42	64	Female	Up to 72 h	1	5	Right	4	+ left	+ left	+ left	−	Not feasible	+	−	+ left
43	67	Male	Up to 72 h	1	1	Left	3	+ right	+ right	+ right	−	Not feasible	−	−	+ right
44	43	Female	72 h up to 3 months	1	1	Right	4	−	+ left	+ left	−	−	−	−	−
45	65	Male	Up to 72 h	1	1	Right	4	+ left	+ left	+ left	−	+	−	−	+ left
46	71	Male	Up to 72 h	1	2	Right	3	+ left	+ left	+ right	−	+	−	−	+ right
47	77	Female	Up to 72 h	1	1	Right	4	−	+ left	+ left	−	+	+	−	+ left
48	62	Male	Up to 72 h	1	3	Right	4	−	−	+ left	−	+	−	−	−
49	74	Female	Up to 72 h	1	1	Right	4	−	+ left	+ left	−	+	−	−	−
50	68	Male	Up to 72 h	1	1	Left	4	+ right	+ right	+ right	−	+	−	−	+ right
51	72	Male	More than 3 months	1	2	Right	4	−	+ left	+ left	+ left	+	−	−	+ both
52	57	Female	Up to 72 h	1	4	Right	4	+ right	+ right	−/+ both	−	Not feasible	−	−	+ right
53	79	Female	Up to 72 h	1	4	Right	4	−	+ left	+ left	−	Not feasible	−	−	+ both
54	73	Male	Up to 72 h	1	1	Left	4	−	+ right	+ right	−	+	−	−	−
55	60	Male	More than 3 months	1	1	Left	4	−	+ right	+ right	−	+	−	−	−
56	54	Male	More than 3 months	1	1	Right	4	−/+ both	+ left	+ left	+ right	Not feasible	−	−	+ both
57	67	Male	Up to 72 h	1	1	Left	4	−	+ right	+ right	−	−	+	−	−
58	73	Male	Up to 72 h	1	1	Right	4	−	+ left	+ left	−	−	+	+	−
59	80	Female	Up to 72 h	1	4	Right	4	+ left	+ left	+ left	+ left	Not feasible	+	−	+ left
60	41	Male	72 h up to 3 months	1	1	Right	4	+ left	+ left	+ left	−	Not feasible	−	−	+ left

Patient's characteristics, categorized by patient number, gender, age, onset of symptoms as well as etiology of symptoms, side of lesion. The graduation of paresis was performed using the oxford scale. According to Medical research council (MRC) scale (Palo Alto 1978; Paternostro‐Sluga et al. 2008). Muscle strength is graded 0 to 5 (3 = through full range actively against gravity, 4 = through full range actively against some resistance, 5 = through full range actively against strong resistance). + = pathological; − = not pathological/unremarkable. Etiology: 1 = ischemia, 2 = hemorrhage, 3 = tumor. Lesion location: 1 = supratentorial mid cerebral artery (MCA), 2* = *supratentorial *anterior* cerebral artery (ACA), 3 = supratentorial posterior cerebral artery (PCA), 4 = infratentorial mesencephalon/midbrain, 5 = infratentorial pons/brainstem, 6 = infratentorial medulla oblongata/brainstem.

BCT, bed cycling test; LHT, leg holding test in supine position.

**Table 4:** Detailed anatomical description of involved structures in patient group

Patient no.	Age	Side of lesion	Graduation of paresis	Anatomical location of lesion
1	81	Right	4	Lateral lenticulostriate artery infarction
2	55	Right	3	Lateral lenticulostriate artery infarction
3	73	Left	3	Post‐ and subcentral media infarction including posterior insula
4	82	Right	4	Lateral lenticulostriate artery infarction
5	82	Left	4	Gyrus postcentralis
6	92	Right	4	Gyrus praecentralis/Sulcus centralis
7	73	Right	3	Medial and lateral lenticulostriate artery infarction
8	66	Right	4	Gyrus praecentralis and postcentralis
9	54	Left	4	Media infarction, superior division
10	78	Right	3	Basal ganglia hemorrhage
11	62	Right	4	Capsula externa and Nucleus caudatus right
12	79	Right	4	Paramedian pons infarction
13	71	Left	4	Putamen and posterior insular region
14	47	Left	4	Corpus ncl. Caudatus
15	55	Left	4	Paramedian ponst infarction
16	86	Left	4	Posterioren Gyrus frontalis medius, Gyrus praecentralis and postcentralis
17	78	Right	4	Lateral lenticulostriate artery infarction
18	62	Left	3	Hemorrhage capsula interna (thalamus/crus posterior)
19	72	Left	4	Corona radiata parietal, temporal (multiple, embolic)
20	87	Right	4	Cortical border zone infarction between MCA und PCA
21	52	Right	4	Basal ganglia hemorrhage
22	38	Right	4	2/3 MCA territory infarction frontal, temporal, insular region and putamen (dorsolateral)
23	69	Right	4	Posterior infarction
24	54	Left	4	Paramedian pons infarction
25	68	Left	4	Infarction of Capsula interna (Crus posterior)
26	78	Left	4	Capsula externa, posterior insula
27	73	Right	3	Anterior Choroideal artery
28	80	Left	4	Anterior Choroideal artery
29	92	Right	3	Lateral lenticulostriate artery infarction
30	81	Left	4	Thalamus
31	34	Right	4	Medulla oblongata (dorsolateral)
32	68	Left	4	MCA‐territory (posterior division)
33	68	Left	4	ACA‐infarction
34	74	Left	4	Capsula interna
35	80	Right	4	Lobus temporalis, basal ganglia, Cortical (MCA territory)
36	93	Right	4	MCA territory, pericentral region, cortical border zone (multiple, embolic)
37	68	Left	4	ACA‐infarction
38	75	Right	4	Corona radiata
39	81	Right	3	Putaman dorsal and posterior insular region
40	21	Right	4	Resection of a meduloblastoma occipital
41	72	Left	4	Basal ganglia
42	64	Right	4	Pons infarction anterolateral
43	67	Left	3	MCA territory (multiple, embolic)
44	43	Right	4	Cortical border zone infarction
45	65	Right	4	Cortical border zone infarction
46	71	Right	3	ACA‐infarction right and PICA
47	77	Right	4	Lacunar infarction basal ganglia
48	62	Right	4	Thalamus medial (PCA territory, multiple embolic)
49	74	Right	4	MCA territory, Gyrus praecentralis, insular region posterior (multiple, embolic)
50	68	Left	4	Lateral lenticulostriate artery infarction (multiple, embolic)
51	72	Right	4	Lacunar infarction white matter media territory
52	57	Right	4	Paramedian pons infarction
53	79	Right	4	Basal ganglia
54	73	Left	4	MCA territory (posterior insular region, basal ganglia, multiple, embolic)
55	60	Left	4	Capsula interna
56	54	Right	4	Lateral and medial lenticulostriate artery infarction (basal ganglia)
57	67	Left	4	Cortical border infarction
58	73	Right	4	Gyrus praecentralis (multiple embolic)
59	80	Right	4	Thalamus dorsolateral
60	41	Right	4	MCA territory (Gyrus praecentralis, postcentralis, Lobus parietalis super, insular region, operculum frontal and temporal, corpus caudatus)

MCA, mid cerebral artery; ACA, anterior cerebral artery; PCA, posterior cerebral artery.

### Control group

Sixty‐two healthy control persons were enrolled, two had to be excluded for technical reasons, 60 subjects were analyzed (38 females, mean age 57.3 ± 18.6). Supplemental material online only shows a control person performing the leg holding test and BCT (see Video S1).

### Sensitivity, specificity, positive predictive value and negative predictive value of the BCT and LHT

The sensitivity and specificity of performing the single execution of each test were 0.64 (95% CI 0.57–0.71) and 0.70 (0.63–0.77) for the BCT only, 0.46 (0.39–0.54), and 0.86 (0.80–0.90) for the LHT only and 0.68 (0.61–0.75) and 0.78 (0.72–0.84) for BCT and LHT together. The positive and negative predictive values were 0.68 (0.60–0.74) and 0.67 (0.60–0.74) for the BCT only, 0.76 (0.67–0.84) and 0.62 (0.56–0.68) for the LHT only and 0.76 (0.69–0.82) and 0.72 (0.65–0.78) for both tests. For details see Table 5. The positive and negative likelihood ratios were 2.2 and 2.0 for the BCT, 3.2 and 1.6 for the LHT, and 3.2 and 2.5 for both tests together.

**Table 5:** Test properties of bed cycling test, leg holding test and performing both clinical tests together. Values are expressed as mean and 95% confidence interval (range)

	BCT	Leg holding test	Both clinical tests
Sensitivity	0.64 (0.57–0.71)	0.42 (0.38–0.54)	0.68 (0.61–0.75)
Specificity	0.70 (0.63–0.77)	0.85 (0.80–0.90)	0.78 (0.72–0.84)
PPV	0.68 (0.60–0.74)	0.76 (0.67–0.84)	0.76 (0.69–0.82)
NPV	0.67 (0.60–0.74)	0.62 (0.57–0.68)	0.72 (0.65–0.78)

BCT: bed cycling test; PPV: positive predictive value; NPV: negative predictive value.

### Comparison of inter‐rater reliability

From the observed numbers of agreements (po) and the Poisson model estimates (beta_diag) Aickin's alpha was computed [alpha = po (1 − e^−beta_diag^) as 0.54, 0.55, and 0.54 for rater groups 1–3].

### Results regarding the different clinical experience

Clinical experience (three categories <2 years, 2–6 years, more than 6 years) did not show a significant effect on the odds of a correct test result. This also held true, when the data set was confined to the BCT only. Compared to a grade 3 paresis the odds for a correct test result was considerably lower for a grade 4 (OR = 0.0385, 95% CI 0.0057–0.1149)), and a grade 5 (OR = 0.1578 (0.0228; 0.4436) paresis. The odds for a correct test result were not significantly higher (1.083, 95% CI 0.7470–1.545) for the BCT than for the LHT, however the OR was 1.653, 95% CI 1.155–2.507 for both tests.

## Discussion

The major findings of this prospective observer‐blinded diagnostic study are that the BCT (63.3%) showed a higher sensitivity compared to the LHT (46.2%), while the LHT was more specific. In the case of a positive LHT, an additional BCT does not provide further valid diagnostic information. In the case of a negative LHT, however, adding a BCT results in a better diagnostic accuracy. The combination of both clinical tests shows the highest sensitivity of 68.4%. In the case of a negative result of the LHT, the additional implementation of the BCT provides further information. This means, that more cerebral lesions can be detected by performing both – the LHT and the BCT. This combination is the most sensitive and time‐efficient method for detecting subtle lesions of the lower limb and could be a very useful and important tool in the evaluation of patients with possible neurological diseases for every emergency physician. The similarity of Aickin's alpha among the rater groups supports the robustness of our results regarding inter‐rater reliability.

In several other recent studies the sensitivity and specificity of known clinical tests have been evaluated. The main emphasis was, however, on the upper limb. A normal leg holding test (Mingazzini test) was reliable to rule out a motor lesions (Teitelbaum et al. 2002). A total of 164 patients who held the maneuver for an average of 10 sec was analyzed showing a sensitivity of 55.4% and specificity of 87.5% (Teitelbaum et al. 2002). Other studies regarding the clinical relevance of the clinical investigation of the lower limb are missing, especially there are no other studies regarding the sensitivity and specificity of the tests. In our study with a total of 120 participants we again could demonstrate the sensitivity (46.20%) and specificity (85.83%) of the leg holding test in supine position – our results are therefore very similar to those described in the literature (Teitelbaum et al. 2002).

One problem of the leg holding maneuver in supine position is that not many patients, especially patients with weak abdominal muscles, are able to perform it properly. In our study we observed that it was easier for patients, especially elderly and obese patients, to perform the BCT.

Of course, we are aware of the limitations of our study: It is quite difficult to blind examiners to the diagnosis in studies on the effectiveness of clinical signs. We attempted this by including a control group using nine different observers with different levels of clinical practice and by showing the BCT, the LHT and both clinical tests to different examiners. The observers were blinded to the history of the patients. However, we cannot rule out the possibility that the observers detected subtle clues that identified patients as having a focal lesion. Moreover, different observers can of course interpret the clinical maneuvers differently.

Secondly, all patients had variable hemiparesis (graded MRC 3 or 4). Moreover, patients with sensory abnormalities that could affect testing were not excluded.

Thirdly, most of our patients with clinical signs had a cerebrovascular disease, only one suffered from a neoplastic focal brain disease. As a result, most of the participants in the patient group had an acute disease.

The BCT mostly detects proximal paresis of the legs as the innervation of the BCT activates proximal muscles of the hip and the leg. While forearm rolling can be considered as more distal motor function, distal paresis of the legs needs further clinical evaluations. Foot tapping test could be a possible addition to the clinical examination. Therefore, the task is performed mostly by distal muscles, which are related to voluntary, discrete and skilled movements (Numasawa et al. 2012). So the importance of distal paresis needs further clinical evaluation. On the other hand, the BCT is a sensitive test for the evaluation of paresis of the lower limb. It is of importance to note that self‐paced voluntary rhythmic movements demand the participation of several central motor and sensory areas. Apart from superficial and deep sensory deficit that could lead to a pathological performance of the BCT, the appearance of foot apraxia or even ataxia may have an impact on this rhythmic movements and therefore to a pathological performance. However, this could be a factor that led to the limited specificity of the BCT.

Finally, it should not be forgotten that in the context of a clinical trial, aspects of the clinical examination and signs could be over‐interpreted. Moreover, the assessment of the “right” graduation of the paresis seems difficult for the evaluators because they can only see the LHT and the BCT.

A strength of the study is the inclusion of raters with different levels of clinical experience from 0.5 to 20 years. The study identified no significant effect of rater experience on the validity of the two tests examined, which makes them simple, non‐invasive, and yet valuable diagnostic tools for clinicians with less experience as well.

## Conclusions

The physical examination is the most important guide in neurology for the further diagnosis of patients with focal neurological deficits. Therefore, we suggest that the clinical examination of the lower limb be expanded to include the BCT on a routine basis in all patients with possible central motor dysfunctions. The implementation of the BCT may also help to detect focal lesions when used by non‐neurologists, but this would need to be analyzed in a separate study.

## Conflict of Interest

K. Feil reports no disclosures. N. Boettcher reports no disclosures. F. Lezius reports no disclosures. M. Habs reports no disclosures. T. Hoegen reports no disclosures. K. Huettemann reports no disclosures. O. Eren reports no disclosures. C. Muth reports no disclosures. F. Schoeberl reports no disclosures. A. Zwergal reports no disclosures. O. Bayer reports no disclosures. M. Strupp is Joint Chief Editor of the Journal of Neurology, Editor in Chief of Frontiers of Neuro‐otology and Section Editor of F1000. He has received speaker's honoraria from Abbott, UCB, GSK, TEVA, Biogen Idec, Pierre‐Fabre, Eisai and Hennig Pharma.

## Supporting information


**Video S1**. Showing a healthy control person performing the leg holding test in the supine position and the bed cycling test.

**Video S2**. Showing patient no. 5 with a paresis on the right site, graduated in clinical examination MRC 4/5.

**Video S3**. Showing patient no. 40 with a paresis on the left site, graduated in clinical examination MRC 4/5.

## References

[brb3445-bib-0001] Amer, M. , G. Hubert , S. J. Sullivan , P. Herbison , E. A. Franz , and G. D. Hammond‐Tooke . 2012 Reliability and diagnostic characteristics of clinical tests of upper limb motor function. J. Clin. Neurosci. 19:1246–1251. 22705136 10.1016/j.jocn.2011.12.007

[brb3445-bib-0002] Anderson, N. E. , D. F. Mason , J. N. Fink , P. S. Bergin , A. J. Charleston , and G. D. Gamble . 2005 Detection of focal cerebral hemisphere lesions using the neurological examination. J. Neurol. Neurosurg. Psychiatry 76:545–549. 15774443 10.1136/jnnp.2004.043679 PMC1739581

[brb3445-bib-0003] Ko, D. Y. , and L. M. Verhagen . 1994 Forearm rolling test. Neurology 44:1364. 8035952 10.1212/wnl.44.7.1364

[brb3445-bib-0004] Koehler, P. J. . 2000 The Barré and Mingazzini test. Oxford University Press, New York.

[brb3445-bib-0005] Nowak, D. A. 2011 The thumb rolling test: a novel variant of the forearm rolling test. Can. J. Neurol. Sci. 38:129–132. 21156442 10.1017/s0317167100011173

[brb3445-bib-0006] Numasawa, T. , A. Ono , K. Wada , Y. Yamasaki , T. Yokoyama , S. Aburakawa , et al. 2012 Simple foot tapping test as a quantitative objective assessment of cervical myelopathy. Spine 37:108–113. 21252819 10.1097/BRS.0b013e31821041f8

[brb3445-bib-0007] Palo Alto, CA . 1978 Medical Research Council of the United Kingdom. Aids to examination of the peripheral nervous system: Memorandum No. 45. Pendragon House, London.

[brb3445-bib-0008] Paternostro‐Sluga, T. , M. Grim‐Stieger , M. Posch , O. Schuhfried , G. Vacariu , C. Mittermaier , et al. 2008 Reliability and validity of the medical research council (mrc) scale and a modified scale for testing muscle strength in patients with radial palsy. J. Rehabil. Med. 40:665–671. 19020701 10.2340/16501977-0235

[brb3445-bib-0009] Sawyer, R. N. Jr , J. P. Hanna , R. L. Ruff , and R. J. Leigh . 1993 Asymmetry of forearm rolling as a sign of unilateral cerebral dysfunction. Neurology 43:1596–1598. 8351018 10.1212/wnl.43.8.1596

[brb3445-bib-0010] Teitelbaum, J. S. , M. Eliasziw , and M. Garner . 2002 Tests of motor function in patients suspected of having mild unilateral cerebral lesions. Can. J. Neurol. Sci. 29:337–344. 12463489 10.1017/s0317167100002201

[brb3445-bib-0011] Yamamoto, T. 1995 Forearm‐rolling test. Neurology 45:2299. 8848218 10.1212/wnl.45.12.2299

